# Coexpressing the Signal Peptide of Vip3A and the Trigger Factor of *Bacillus thuringiensis* Enhances the Production Yield and Solubility of eGFP in *Escherichia coli*

**DOI:** 10.3389/fmicb.2022.892428

**Published:** 2022-07-18

**Authors:** Jianhua Gao, Chunping Ouyang, Juanli Zhao, Yan Han, Qinghua Guo, Xuan Liu, Tianjiao Zhang, Ming Duan, Xingchun Wang, Chao Xu

**Affiliations:** ^1^Shanxi Key Laboratory of Minor Crops Germplasm Innovation and Molecular Breeding, College of Life Sciences, Shanxi Agricultural University, Jinzhong, China; ^2^Experimental Teaching Center, Shanxi Agricultural University, Jinzhong, China; ^3^State Key Laboratory of Rice Biology, Institute of Insect Sciences, College of Agriculture and Biotechnology, Zhejiang University, Hangzhou, China

**Keywords:** Vip3, *Bacillus thuringiensis*, signal peptide, fusion tag, eGFP, *Bt* trigger factor

## Abstract

Many fusion tags have been developed to improve the expression of recombinant proteins. Besides the translocation of cargo proteins, the signal peptides (SPs) of some secretory proteins, such as the ssTorA and Iasp, have been used as an inclusion body tag (IB-tag) or the recombinant expression enhancer in the cytosol of *E. coli*. In this study, the approach to utilize the SP of Vip3A (Vasp) from *Bacillus thuringiensis* (*Bt*) as a fusion tag was investigated. The results showed that either the Vasp or its predicted N- (VN), H- (VH), and C-regions (VC), as well as their combinations (VNH, VNC, and VHC), were able to significantly enhance the production yield of eGFP. However, the hydrophobic region of the Vasp (VH and/or VC) made more than half of the eGFP molecules aggregated (VeGFP, VHeGFP, VCeGFP, VNHeGFP, VNCeGFP, and VHCeGFP). Interestingly, the addition of the *Bt* trigger factor (*Bt*TF) led to the neutralization of the negative impact and solubilization of the fusion proteins. Therefore, the coexpression of Vasp or its derivates with the chaperone *Bt*TF could be a novel dual-enhancement system for the production yield and solubility of recombinant proteins. Notably, *Ec*TF was unable to impact the solubility of Vasp or its derivates guided proteins, suggesting its different specificities on the recognition or interaction. Additionally, this study also suggested that the translocation of Vip3 in the host cell would be regulated by the *Bt*TF-involved model.

## Introduction

Fusion tags have been widely used for producing recombinant proteins. A fusion tag can be derived from an artificial polypeptide, a partial fragment, or a whole natural protein. Many fusion tags have been applied to facilitate protein purification or improve protein production yield, solubility, and folding (reviewed in Costa et al., 2014; Rosano and Ceccarelli, 2014; Paraskevopoulou and Falcone, 2018; Vandemoortele et al., 2019; Ki and Pack, 2020). Some of them are versatile. For instance, the well-known solubility enhancer tags such as maltose-binding protein (MBP) (Maina et al., 1988), glutathione S-transferase (GST) (Smith and Johnson, 1988), small ubiquitin-related modifier (SUMO) (Marblestone et al., 2006), and Fasciola hepatica 8-kDa antigen (Fh8) (Costa et al., 2013) are also used in affinity purification. Signal peptides (SPs) of many secretory proteins have also been used as fusion tags to direct the recombinant proteins to different cellular locations (reviewed in Overton, 2014; Rosano and Ceccarelli, 2014; Gupta and Shukla, 2016; Malik, 2016; Kleiner-Grote et al., 2018). Recently, novel applications of the SP tags were developed. For instance, the SP of trimethylamine N-oxide reductase (torA) in *E. coli* was reported to be an inclusion body tag (IB-tag) that produced some recombinant proteins in insoluble form in *E. coli* cytosol (Jong et al., 2017). The SP of Cry1Ia toxin (Iasp) of *Bacillus thuringiensis* (*Bt*) can not translocate recombinant proteins through the cell membrane in high efficiency but enhance their expression level significantly (Gao et al., 2020). The produced fusion fluorescent indicators such as IeGFP and ImCherry showed better performance than the individually expressed eGFP and mCherry in *E. coli* and *Bt* strains. Notably, SPs are ubiquitous in nature and are worthy of deeper exploration.

Vip3 toxins were produced by *Bt* cells during the vegetative stage of growth. The first *vip3* gene was reported in 1996, and since then, many homologs were collected (Crickmore et al., 2020). At present, the action mode of the Vip3 toxins still remains elusive. Two ways were proposed to solve the problem: apoptosis induced by the Vip3A protoxin and cell perforation mediated by the activated Vip3A toxin (reviewed in Chakroun et al., 2016; Chakrabarty et al., 2020; Syed et al., 2020). Several pieces of research concluded that there was no significant cross-resistance between the Vip3 and Cry families, suggesting the different receptors for both toxins when acted inside the target insects (reviewed in Chakroun et al., 2016; Chakrabarty et al., 2020; Syed et al., 2020). Recently, the determination of the three-dimensional structure (3D-structure) of the protease-activated Vip3A and Vip3B at higher resolution revealed a homotetramer architecture containing a long coiled-coil that explained the structural basis of the perforation mode (Núñez-Ramírez et al., 2020; Zheng et al., 2020). Vip3 proteins share a similar overall tetrameric organization, especially for the domains I, II, and III at the N terminus, but the last two domains (IV and V) show slight variation in position and orientation that would result from the greater diversity of the primary structure at the C-terminus of the Vip3 family (Chakroun et al., 2016).

The N terminus of Vip3, including the predicted SP region (Vsp), was conserved (Chakroun et al., 2016). Due to the deficiency of the peptidase recognition site, the exact length of Vsp is still unknown (Estruch et al., 1996; Doss et al., 2002; Chen et al., 2003; Rang et al., 2005). The N-terminal 33 amino acids (AAs) are the longest version of Vsp (Rang et al., 2005) in which three classic regions can be roughly distinguished, i.e., the positively charged N-region (from M1 to R11), followed by the hydrophobic residues (H-region, from A12 to F20), and the C-region (from N21 to I33). It is noteworthy that the C-region is rich in hydrophilic AAs, but the counterpart of Vsp has a highly amphiphilic nature resulting from the alternative arrangement of the hydrophilic and hydrophobic AAs. The alternative arrangement in this region facilitates the formation of a four-helix bundle, transporting protons and divalent metals through the membrane, by contacting with the lipid bilayer of targeted cells in the final architecture of Vip3 tetramer (Núñez-Ramírez et al., 2020).

Interactions between the nascent peptide and the signal peptide recognition proteins or the molecular chaperones prevent the recombinant protein from aggregation, thus facilitating the translocation. At present, the translocation pathway of Vip3 is still unclarified. Given that the strongly conserved motif (S/T-RRXFLK) in SP of the twin-arginine translocation system (Tat system) is not present in Vsp, Vip3 proteins might translocate *via* the other single membrane-spanning secretion system, the Sec pathway (reviewed in Berks, 2015; Costa et al., 2015; Green and Mecsas, 2016; Anné et al., 2017). In contrast to most secretory proteins, Vsp is not removed after Vip3 protein translocation. The hydrophobic regions in SPs would interact with the corresponding regions of the client proteins and therefore negatively affect its folding. For instance, the existence of SP of MBP or pelB (pectate lyase B) at the N-terminus interfered with the thermodynamic stability of mature MBP or thioredoxin (Trx) (Beena et al., 2004; Krishnan et al., 2009; Kulothungan et al., 2009; Singh et al., 2013). This observation would explain the capability of inducing inclusion body formation of the SPs described above (Jong et al., 2017). This study will explore the effects of the predicted SP of Vip3A protein (Vasp) and its derivates on the expression of enhanced green fluorescent protein (eGFP) and provide a novel solution for enhancing the production yield and solubility of recombinant proteins, simultaneously.

## Materials and Methods

### Bacterial Strains and Growth Conditions

All plasmids and strains used in this study are listed in Supplementary Table S1. Unless otherwise noted, the *E. coli* cells were incubated in Luria-Bertani medium (LB medium) with 50 μg/ml ampicillin for pMD19, pHT304 and its derived vectors, or 50 μg/ml kanamycin for pET28a and its derived vectors at 37°C shaking with 200 rpm.

### Sequence Analysis of the SPs of Vip3 Proteins

For phylogenetic analysis, the Vip3 protein sequences were retrieved from Bacterial Pesticidal Protein Resource Center (https://www.bpprc.org/) for extracting the corresponding SP sequences (Crickmore et al., 2020). Then, the peptide alignment was conducted using MAFFT software (v7.453) (Katoh and Standley, 2013) with default parameters, and the result was graphically enhanced by ESPript (v3.0) (Robert and Gouet, 2014).

The hydropathicity scale of the SP sequences of Vip3Aa1, MBP, pelB, and TorA was computed by the online program ProtScale (https://web.expasy.org/protscale/) with the default parameters except that the scale values were normalized (Kyte and Doolittle, 1982). The output was plotted by GraphPad Prism (v9.0.0).

### Construction of Expression Vectors

#### pET Expression Constructions

The Vasp-encoding sequence (primers VEGFP-F and VEGFP-fuR) was fused to the 5′ end of the *egfp* gene (primers VEGFP-fuF and I/EGFP-R) by overlapping PCR using primers VEGFP-F and I/EGFP-R to produce the *Vegfp* gene. The *Vegfp* gene was TA-cloned into the pMD19 vector (Takara, Beijing, China) for sequencing. The correct *Vegfp* sequence was cloned into the *Bam*H I/*Xho* I restriction site of the pET28aDel plasmid to form p28aD-VeGFP. Then, the Vasp-encoding sequence in p28aD-VeGFP was replaced by the synthesized fragments to produce the p28aD-VNeGFP, p28aD-VNHeGFP, p28aD-VNCeGFP, p28aD-VHeGFP, p28aD-VHCeGFP, and p28aD-VCeGFP. The *BtCsaA* promoter (*P*_*BtCsaA*_) guided chaperone genes encoding SecA (*Bt*SecA), TF (*Bt*TF), and CsaA (*Bt*CsaA) of *Bt* and SecB of *E. coli* (*Ec*SecB) as well as its mutants (SecB7577, SecB142, and SecB142-7577) were artificially synthesized and cloned into the *Xho* I/*Blp* I site of the p28aD-VeGFP vector. The *P*_*BtCsaA*_-*Bttig* sequence was also inserted into the same site of the p28aD-VNeGFP, p28aD-VNHeGFP, p28aD-VNCeGFP, p28aD-VHeGFP, p28aD-VHCeGFP, and p28aD-VCeGFP, respectively.

The genes that include human *growth differentiating factor*-8 (*GDF8*), *Setaria italica cytochrome P450 71A1*-like (*71A1*), *ent-Cassadiene C2-hydroxylase* (*ECH*), and *cinnamate beta-D-glucosyltransferase* (*CGT*) were artificially synthesized and cloned into the pET28aDel derived plasmid containing *P*_*BtCsaA*_-*Bttig* sequence.

These plasmids were individually transformed into *E. coli* BL21-star (DE3) strain by calcium chloride (CaCl_2_) transformation.

#### pHT304 Expression Constructions

The *Vegfp* gene was amplified by PCR using the p28aD-VeGFP plasmids as the template (primers VEGFP-F and 304I/EGFP-R) and was TA-cloned into the pMD19 vector. After sequencing, the *Bam*H I/*Sac* I fragment of the *Vegfp* gene was inserted into the corresponding site of the pAc-eGFP vector. The plasmid was designated as pAc-VeGFP and was transformed into *E. coli* MC4100 strain.

### Proteins Expression and Samples Preparation

#### *E. coli* MC4100 Strain

The recombinant protein expression protocol in *E. coli* MC4100 was the same as in *E. coli* TG1 that was described previously (Gao et al., 2020). Briefly, the single colony of each *E. coli* strain harboring p304ΔSacI or its derived vectors was inoculated into 3 ml LB medium and incubated overnight. Each strain was inoculated into 8 ml fresh LB medium with an equally initial amount. Three replicates were tested for each strain. At 8 or 12 h after inoculation, 3 ml of cell cultures for each replicate was taken for protein analysis.

#### *E. coli* BL21-Star (DE3) Strain

The overnight incubated cells of each BL21-star (DE3) strain in 3 ml LB medium were transferred into fresh LB medium (1:1,000, v/v) and continuously cultivated in the same condition. IPTG (isopropyl β-D-1-thiogalactopyranoside, 1 mM or other concentrations indicated) was used to induce the recombinant protein expression when the optical density at 600 nm (OD_600_) of the cell culture reached 0.8. After induction at 16°C, the cells of each sample were harvested after centrifuging at 8,000 rpm. The collected cells were washed with deionized water three times and resuspended in 2/5 of the initial volume of ice-cold PBS buffer (137 mM NaCl, 2.7 mM KCl, 10 mM Na_2_HPO_4_, and 2 mM KH_2_PO_4_, pH 7.4). The resuspended cells were disrupted by ultrasonic treatment in ice-cold conditions. A fraction of the cell lysate was pipetted into a new centrifuge tube (total protein sample). After centrifuging at 12,000 rpm at 4°C for 15 min, the remaining lysates were separated. The precipitate was resuspended by the same volume of PBS buffer as the corresponding supernatant. These samples were prepared for sodium dodecyl sulfate–polyacrylamide gel electrophoresis (SDS-PAGE) analysis.

### SDS-PAGE Analysis

The sample preparation and SDS-PAGE analysis referred to the method described previously (Gao et al., 2020). Briefly, each protein sample was mixed with one-fourth volume of 5× SDS gel-loading buffer and boiled for 5 min. The samples were centrifuged at 12,000 rpm for 5 min and then loaded onto SDS-PAGE gels for separation. The separated proteins were stained by Coomassie bright blue and the gels were photographed and analyzed by Bio-Rad Image-Lab-Software (v6.0.1). For western blot analysis, the separated proteins in the gel were transformed to the nitrocellulose membrane and then incubated with the rabbit antiserum against IeGFP protein and the horseradish peroxidase-conjugated goat antirabbit IgG (H + L) antibody (MultiSciences, Hangzhou, China) successively. The target bands were visualized using eECL western blot kit (Cowin Biotech, Jiangsu, China).

### Fluorescence Localization

The *E. coli* cells were prepared by the method described previously (Gao et al., 2020). Briefly, the harvested cells corresponding to 1 A600 unit were washed two times by PBS buffer and immersed in 600 μl of the fixing solution (2% paraformaldehyde, 2.5% glutaraldehyde in PBS buffer). After 45-min incubation at room temperature, the cells were washed three times with PBS and finally resuspended in 100 μl PBS buffer. The suspension was detected by an inverted confocal microscope (Leica SP8, Leica Microsystems, Wetzlar, Germany) with a 63-time oil immersion objective. The outputs were recorded with Leica Application Suite X (v3.1.5).

## Results

### Sequence Conservation and Hydrophobicity Scale of Vsp

**Figure 1:**
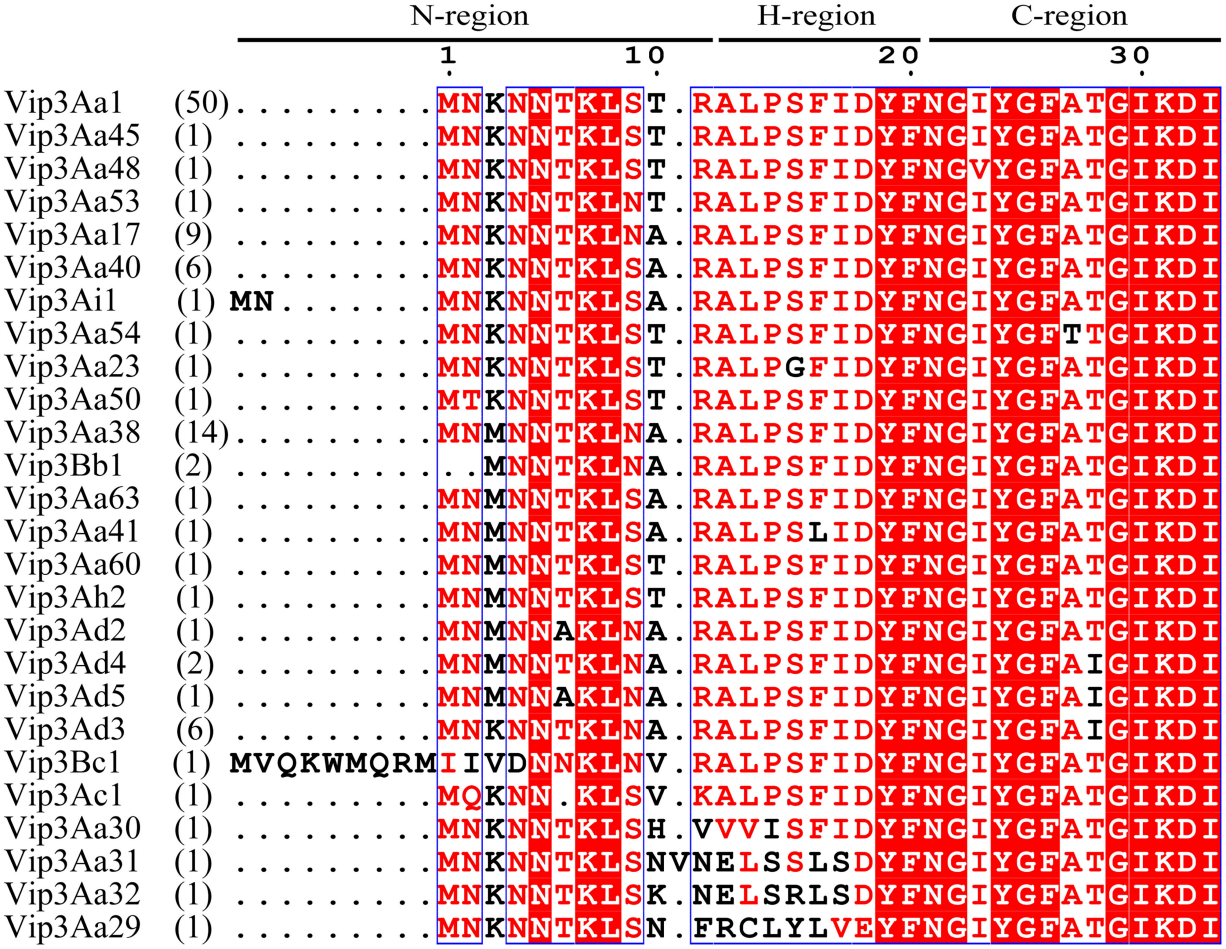
The alignment of the representative Vsp sequences. The predicted N-, H-, and C-regions of SP are indicated. The numbers of genes sharing the corresponding sequences are indicated in parentheses.

A total of 108 Vip3 proteins were collected in the Bacterial Pesticidal Protein Resource Center (Crickmore et al., 2020), but only 26 patterns were found in the predicted Vsp region (Figure 1; Supplementary Table S3). For instance, there were 49 Vip3 proteins that shared the same AA sequences with Vip3Aa1 at N-terminus. The alignment results indicated the high conservation of Vsps, especially in the predicted C-region, which is also the part of helix α1 (from I23 to K39) of Vip3 protein (Núñez-Ramírez et al., 2020). It was observed that three Vip3 proteins, including Vip3Aa31, Vip3Aa32, and Vip3Aa29, were more divergent in the predicted H-region compared to other members. The nine AAs extensions were observed in the predicted N-region of Vip3Bc1, whereas only two extra AAs were found in Vip3Ai1.

**Figure 2:**
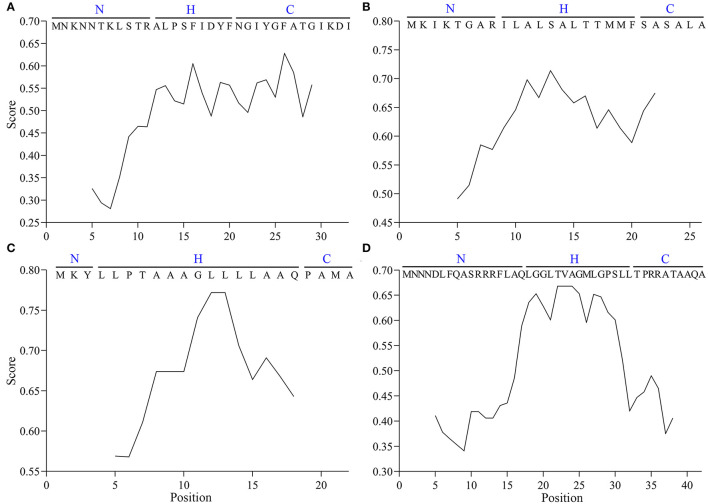
The hydrophobicity scale of SPs of Vip3A **(A)**, MBP **(B)**, pelB **(C)**, and TorA **(D)** proteins.

Given the high frequency of the Vip3Aa1 SP sequence in the Vip3 family, it was considered a representative pattern and designated as Vasp in this study. The hydrophobicity scale of Vasp was determined, and the differences with the common SPs of MBP, pelB, and TorA were revealed (Figure 2). The classic distribution of AA residues in the latter three SPs, including the positively charged N-region, the hydrophobic H-region, and the hydrophilic C-region, was observed. However, in Vasp, the H- and C-regions were ambiguous in which the hydrophilic and hydrophobic AAs were arranged alternatively, resulting in a relatively amphiphilic nature (Núñez-Ramírez et al., 2020). Notably, the hydrophobicity level of the H- and C-regions (0.45–0.65 in Figure 2A) matched with the H-region of the MBP (0.55–0.75 in Figure 2B), pelB (0.55–0.80 in Figure 2C), and TorA's (0.55–0.70 in Figure 2D) SPs, respectively.

### Vasp Enhanced the Expression Level of eGFP in *E. coli* MC4100 Strain

**Figure 3:**
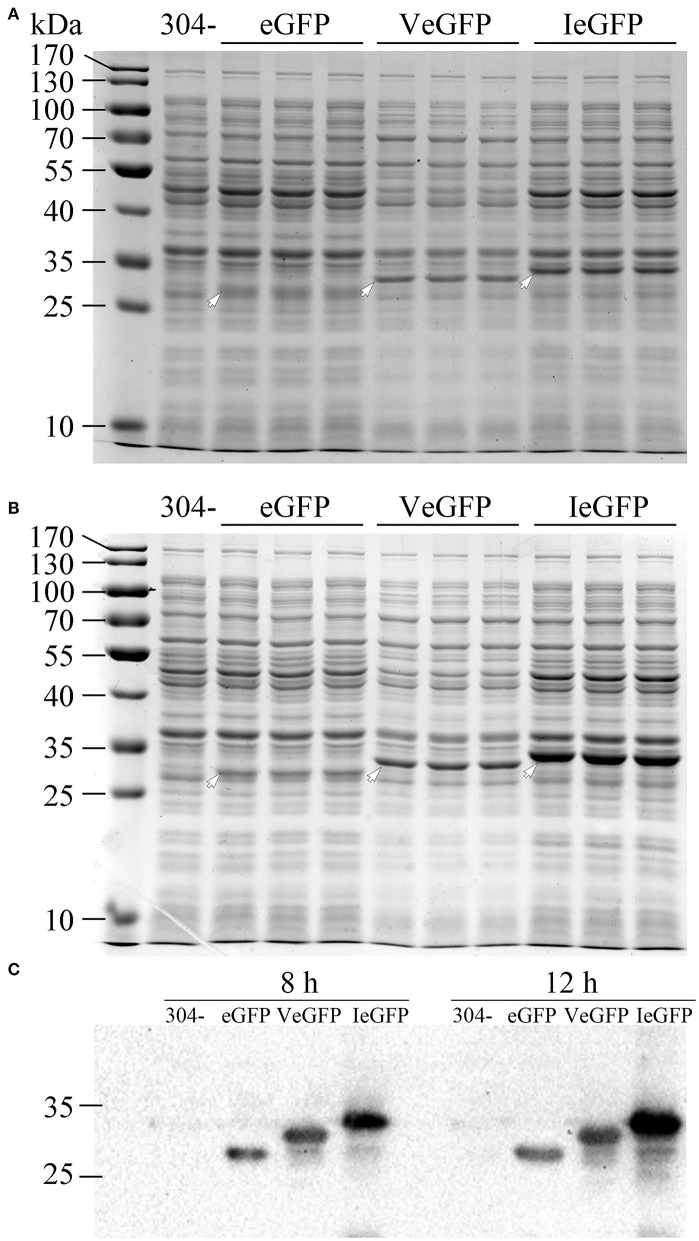
The expression of VeGFP in the *E. coli* MC4100 strain controlled by the constitutive promoter *P*_*ac*_. The samples of eGFP (27.9 kDa), VeGFP (lane 2, 31.8 kDa), and IeGFP (lane 3, 33.1 kDa) were prepared at 8 **(A)** and 12 h **(B)** after inoculation, respectively. Lane “–” is the negative control prepared from M304 cells. Lane “M” is the molecular weight standard. The arrows indicate the target bands of the recombinant proteins. The target proteins were also verified by western blot **(C)**.

The effect of Vasp on the eGFP protein (VeGFP) was investigated using the constitutive promoter of the *cry1Ac* gene (*P*_*ac*_) in *E. coli* MC4100 cells. The expression cassette of the *Vegfp* gene was similar to the *Iegfp* described previously (Gao et al., 2020). The results showed that the expression level of the eGFP was improved slightly by Vasp at 8 (~1.14-folds) and 12 h (~2.05-folds) after inoculation (Figure 3). The observation prompts that Vasp would be also used as another production-enhancement fusion tag such as Iasp.

### *Bt* Trigger Factor Improved the Solubility of the VeGFP

**Figure 4:**
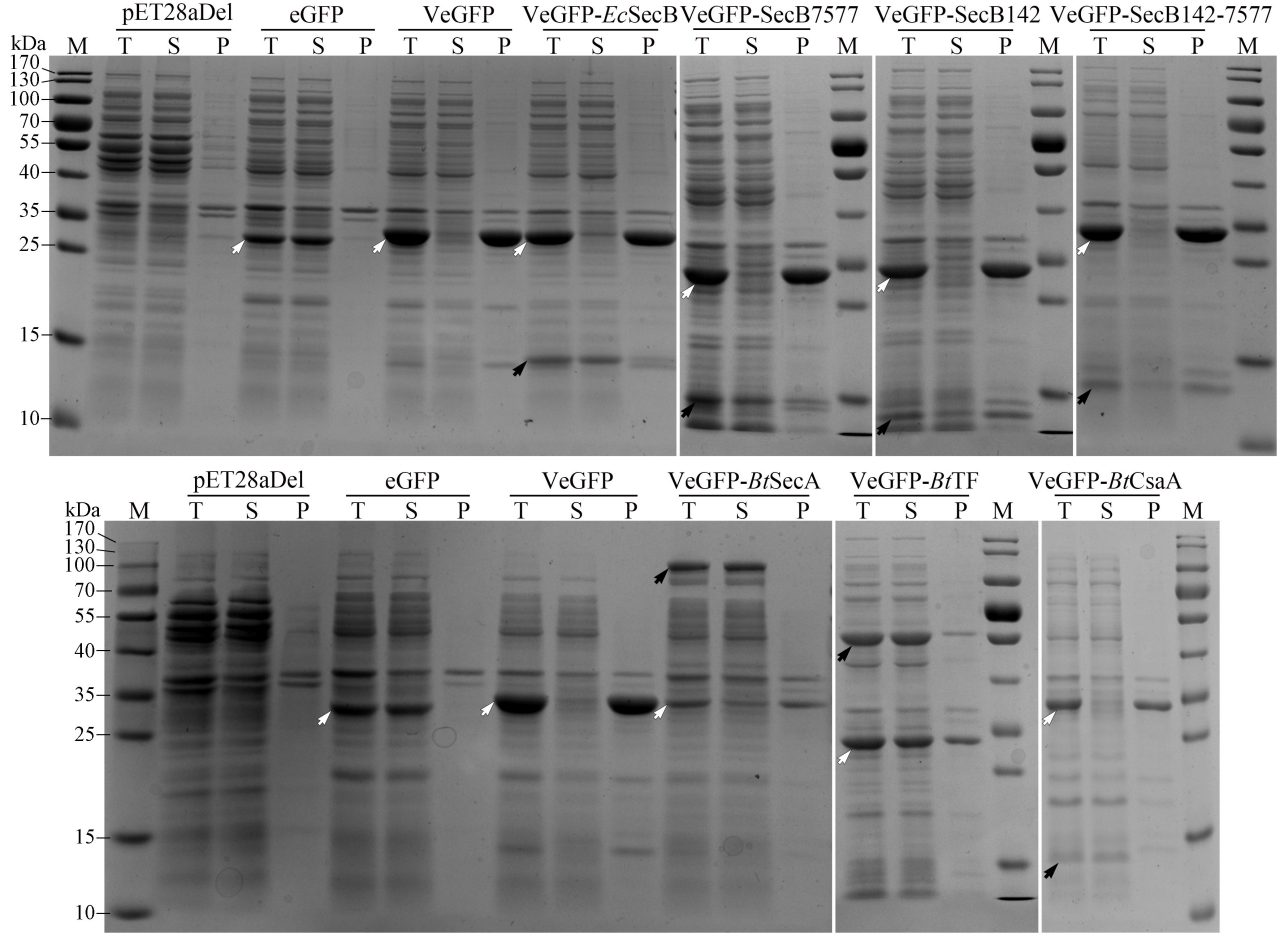
Coexpression analysis of VeGFP with several chaperones. For each sample, the total proteins (T), the soluble component after cell lysis (S), and the precipitates (P) were loaded, respectively. The black arrows indicate the corresponding chaperones (*Ec*SecB, 17.3 kDa; SecB7577, 17.3 kDa; SecB142, 15.9 kDa; SecB142-7577, 15.9 kDa; *Bt*CsaA, 11.9 kDa; *Bt*SecA, 95.0 kDa; *Bt*TF, 47.3 kDa). The hollowed arrows indicate the target recombinant proteins.

Previously, the reservation of SP was reported to affect the thermodynamic stability of the client protein, such as MBP and Trx (Beena et al., 2004; Krishnan et al., 2009; Kulothungan et al., 2009; Singh et al., 2013). In this study, the negative impact of Vasp on the solubility of eGFP was also observed in the BL21-star (DE3) strain (Figure 4), i.e., only 2.5% of products were soluble (Table 1). Since the Tat-pathway was excluded according to the conserved motif analysis, the performances of several chaperones in the Sec pathway in the *Bt* strain including *Bt*SecA, *Bt* trigger factor (*Bt*TF), and also *Bt*CsaA and its counterpart in *E. coli* (*Ec*SecB) on the aggregation-prone protein VeGFP were investigated. Each chaperone gene is located at the 3′ end of the *BtCsaA* promoter (*P*_*BtCsaA*_) that follows the *Vegfp* gene directly (Supplementary Figure S1A). Controlled by the regulation structure, the expression of the VeGFP and *Bt*TF was detected simultaneously after the IPTG induction (Supplementary Figure S1B). The results showed that both *Bt*CsaA and its counterpart *Ec*SecB were unable to prevent the VeGFP from aggregation (Figure 4). *Bt*SecA, a translocation ATPase in Sec pathway, also failed to keep the VeGFP soluble. To exclude the possible interference resulting from the interaction between *Ec*SecB and *Ec*SecA, the variants of *Ec*SecB were tested. *Ec*SecB mutant (SecB7577) with substitutions at two positions, L75Q and E77V, still bound the client proteins but showed a marked reduction in its binding affinities for *Ec*SecA, leading to the disruption of translocation (Fekkes et al., 1998; Sala et al., 2017). The 13 AAs at the C-terminus decreased the dimerization efficiency of SecA (Randall et al., 2005). In this study, the SecB7577, the 13 AAs deficiency mutant at C-terminus (SecB142), and even their combination SecB142-7577 did not impact the solubility of VeGFP positively. Interestingly, only the chaperone *Bt*TF made VeGFP solubilized.

**Table 1:** The soluble fraction of target proteins in the *E. coli* BL21-star (DE3) strain.

**Protein**	**Relative content**				**Mean +**	**Fold**
	**of replicates (%)**				**SEM (%)**	**change**
	**1**	**2**	**3**	**4**		
VeGFP	2	2	3	3	2.5 ± 0.0	25.00**
VeGFP-*Bt*TF	55	55	71	69	62.5 ± 4.0	
VNeGFP	82	65	62	89	74.5 ± 7.0	1.34**
VNeGFP-*Bt*TF	100	100	100	100	100.0 ± 0.0	
VHeGFP	82	56	88	76	75.5 ± 7.0	1.26
VHeGFP-*Bt*TF	100	100	82	100	95.5 ± 5.0	
VCeGFP	53	77	81	68	69.8 ± 6.0	1.26
VCeGFP-*Bt*TF	78	99	83	92	88.0 ± 5.0	
VNHeGFP	53	54	42	46	48.8 ± 3.0	1.96**
VNHeGFP-*Bt*TF	95	92	96	99	95.5 ± 1.0	
VNCeGFP	49	45	45	51	47.5 ± 2.0	2.03**
VNCeGFP-*Bt*TF	97	96	96	97	96.5 ± 0.0	
VHCeGFP	15	13	17	14	14.8 ± 1.0	5.70**
VHCeGFP-*Bt*TF	81	90	82	84	84.3 ± 2.0	

The double asterisks (^**^) indicate the significance of the difference (p <0.01) of soluble fraction w/o BtTF.

**Figure 5:**
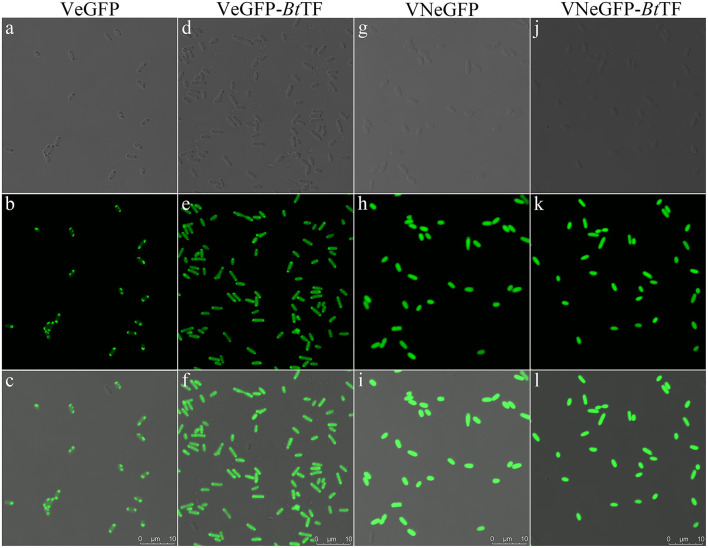
The fluorescent signal distribution of VeGFP and VNeGFP in *E. coli* BL21-star (DE3) cells. The corresponding cells expressing VeGFP **(a–c)** or VNeGFP **(g–h)** individually, or with *Bt*TF **d–f** for VeGFP and **j–l** for VNeGFP) were observed using an inverted confocal microscope (Leica SP8). For each sample, the image in the third row was merged with the corresponding bright-field image in the first row and the fluorescent one in the second row. The scale bar represents 10 μm.

The polar localization of VeGFP products in the BL28-VeGFP strain was observed by confocal microscope (Figures 5a–c). The light spots in each cell indicated the corresponding inclusion body cores (Rinas et al., 2017). However, when expressed together with *Bt*TF (BL28-VeGFP-*Bt*TF strain), the clear fluorescent signal inside the cell emerged evenly without the disappearence of the polar bright spots (Figures 5d–f). The observations were in line with the SDS-PAGE analysis and suggested that the accumulation of *Bt*TF made part of the VeGFP products solubilized and folded correctly in the cytosol.

### Three Regions of Vasp Boosted the Production Yield of eGFP With Distinct Solubility

**Figure 6:**
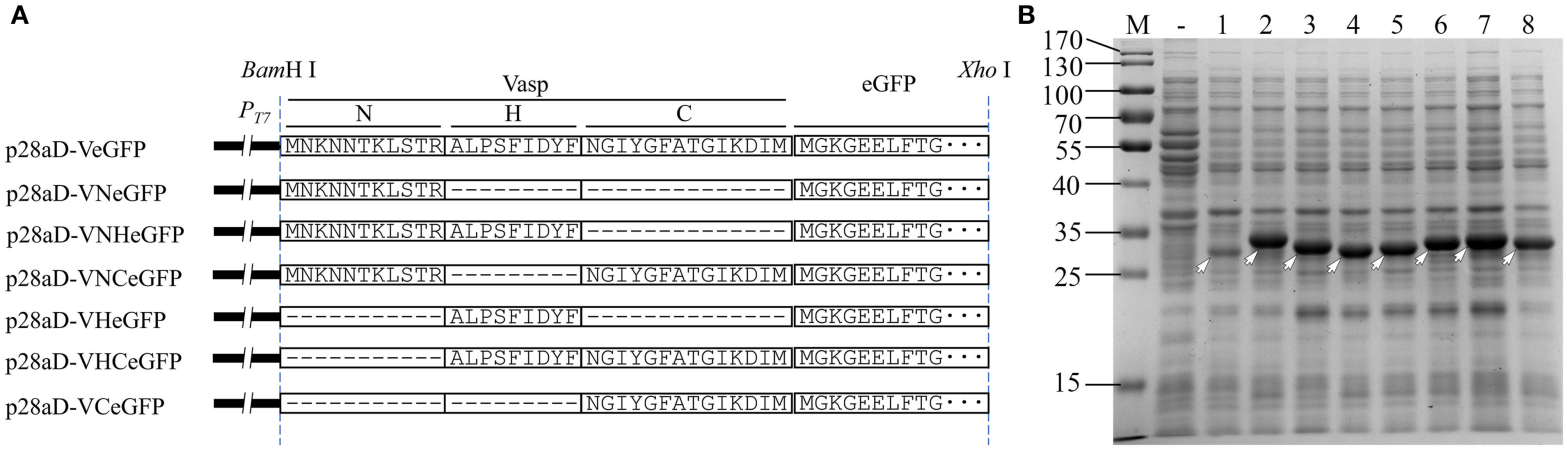
Diagram of Vasp variants **(A)** and their effect **(B)** on the expression of eGFP. In panel **B**, the target bands of eGFP (lane 1), VeGFP (lane 2), VNeGFP (lane 3, 29.2 kDa), VHeGFP (lane 4, 29.1 kDa), VCeGFP (lane 5, 29.5 kDa), VNHeGFP (lane 6, 30.3 kDa), VNCeGFP (lane 7, 30.7 kDa), and VHCeGFP (lane 8, 30.6 kDa) are indicated by hollowed arrows, respectively. Lane “–” is the negative control prepared from BL28aD cells. Lane “M” is the molecular weight standard.

**Figure 7:**
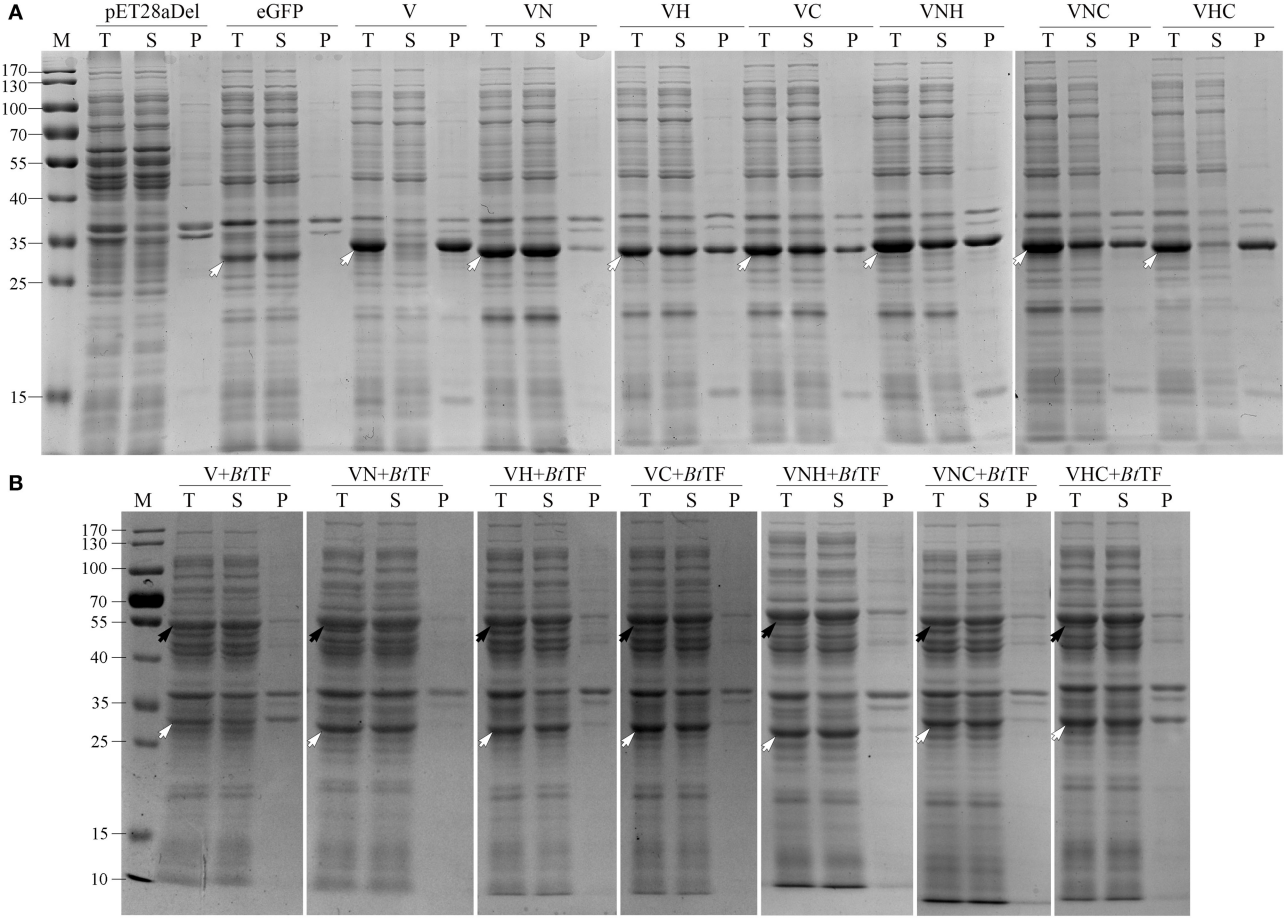
The solubility analysis of the fusion fluorescent proteins with **(A)** or without **(B)** the coexpression of *Bt*TF. For each sample, the total proteins (T), the soluble component after cell lysis (S), and the precipitates (P) were loaded. The black arrows indicate the target bands of *Bt*TF and the hollowed arrows indicate the target recombinant proteins including eGFP, VeGFP (V), VNeGFP (VN), VHeGFP (VH), VCeGFP (VC), VNHeGFP (VNH), VNCeGFP (VNC), and VHCeGFP (VHC). Lane “M” is the molecular weight standard.

To confirm the crucial segment enhancing the expression level of eGFP in Vasp, the predicted N- (VaspN, VN), H- (VaspH, VH), and C-regions (VaspC, VC), as well as their combinations, were used to guide the fluorescent protein, respectively (Figure 6A). As a result, all of these extra peptides boosted the production yield of the eGFP (~2.49- to 3.20-fold enhancement compared to eGFP, Figure 6B; Supplementary Table S4). However, their solubility varied widely (Figure 7A; Table 1). For instance, deletion of H- (VNCeGFP, 47.5%) or C-region (VNHeGFP, 48.8%) both recovered the solubility partially of VeGFP (2.5%). The individual H- or C-region produced 75.5 or 69.8% soluble recombinant proteins (VHeGFP and VCeGFP), respectively. When both the hydrophobic regions were removed (VNeGFP), most of the product molecules (74.5%) were also kept soluble, and even distribution of the fluorescent signal was observed inside the cells (Figures 5g–i). The loss of the N-region (VHCeGFP) led to 14.8% of the products being soluble. All of these results indicated the negative effects of the extra fragments originated from Vasp on the solubility of the client protein. These data revealed the negative correlation between the sequence integrity of Vasp and the solubility of the corresponding fusion fluorescent proteins, and interestingly, the decrements of the solubility impacted by the combination of VN with any one of the other two regions (VH or VC) were cumulative. Approximately a 51.2% decrease in VNHeGFP solubility would result from the addition of the impact of VN (25.5%) and VH (24.5%). Surprisingly, the synergetic effects were observed when the VH and VC coexisted. Approximately 85.2% insoluble fraction is far more than the cumulative effect of the VH (24.5%) and VC (30.2%). A similar result was observed on the maximal reduction in solubility for VeGFP (97.5%) compared to the cumulative effect of the three individual regions.

### *Bt*TF Improved the Solubility of the Fusion Fluorescent Proteins

The coexpressed chaperone *Bt*TF also made the variants of VeGFP soluble (Figure 7B; Table 1). The soluble fraction of VeGFP coexpressed with *Bt*TF (VeGFP+*Bt*TF) increased by 25 fold (2.5 vs. 62.5%). Similarly, the solubility of VHCeGFP, VNHeGFP, VNCeGFP, VHeGFP, and VCeGFP proteins expressed in the *E. coli* BL21-star (DE3) strain was improved by 5.70-, 1.96-, 2.03-, 1.26-, and 1.26-fold by *Bt*TF, respectively. Interestingly, the *Bt*TF was able to make all of the tested VNeGFP molecules soluble (1.34-folds, Figures 5j–l, 7B; Table 1). These data also indicated the positive correlation between the sequence integrity of Vasp and the interaction strength with *Bt*TF.

### The Novel Expression System Was Also Applicable to Other Recombinant Proteins

**Figure 8:**
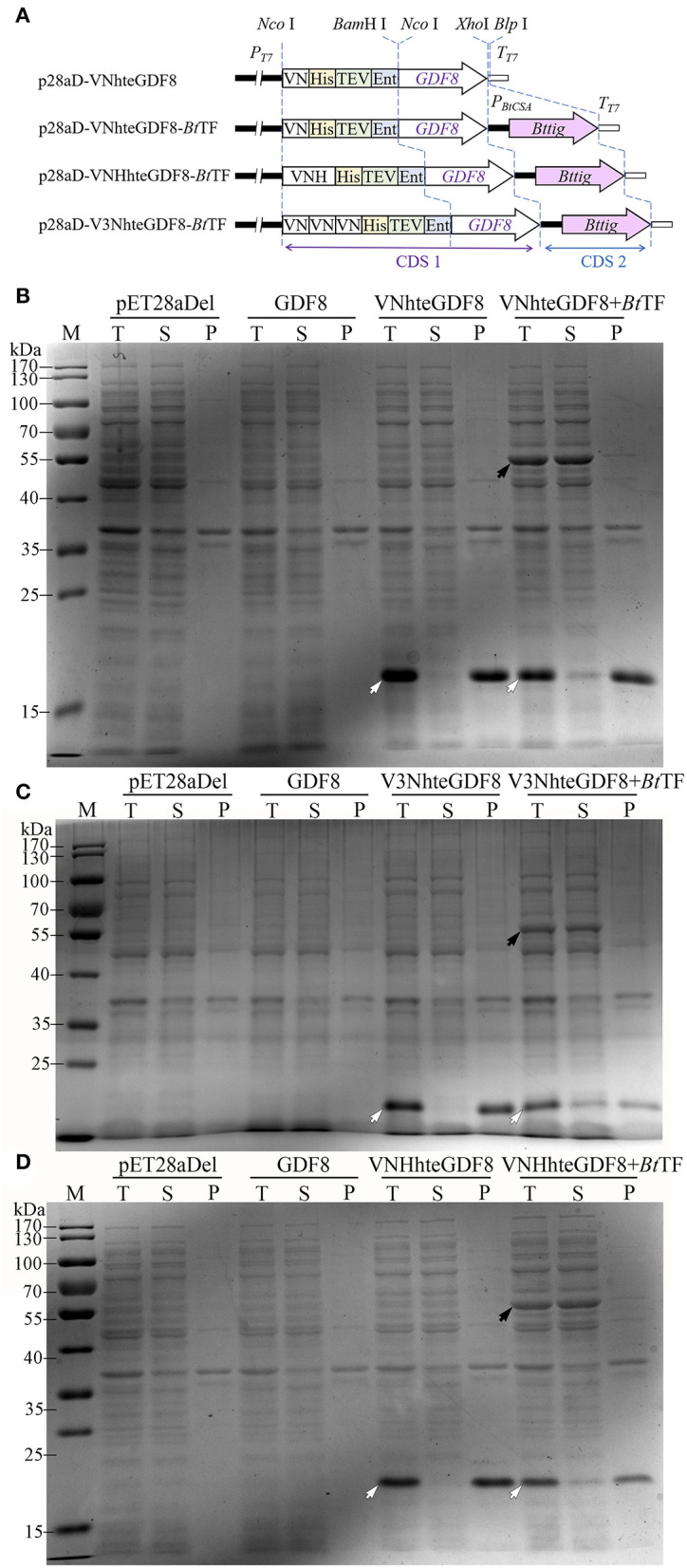
The effect of the Vasp derivates and *Bt*TF on the expression of GDF8 protein. **(A)** The diagram of expression cassettes of VNhteGDF8, VNHhteGDF8, and V3NhteGDF8. The expression of VNhteGDF8 (**B**, 16.9 kDa), V3NhteGDF8 (**C**, 19.2 kDa), and VNHhteGDF8 (**D**, 18.0 kDa) was analyzed by SDS-PAGE. For each sample, the total proteins (T), the soluble component after cell lysis (S), and the precipitates (P) were loaded. Lane “M” is the molecular weight standard. The black arrow indicates the *Bt*TF and the hollowed arrow indicates the recombinant proteins.

According to the results described above, the combination of *Bt*TF and Vasp or its variants would be used as a novel expression system. The expression vectors were redesigned for adding the encoding sequences of His-tag, TEV protease, and enterokinase recognition sites at the 3′ end of VN or VNH-encoding sequences (Figure 8A, p28aD-VNhteGDF8-*Bt*TF and p28aD-VNHhteGDF8-*Bt*TF). The expression cassette structures were designated as the VNhte-*Bt*TF or VNHhte-*Bt*TF, respectively. Additionally, three VN-encoding sequences were arranged in a tandem array in the p28aD-V3NhteGDF8-*Bt*TF plasmid (the V3Nhte-*Bt*TF cassette). The optimum concentration of IPTG (0.05 mM) for the novel vectors was identified by expressing the VNhteGDF8 protein in the *E. coli* BL21-star (DE3) strain harboring the p28aD-VNhteGDF8-*Bt*TF plasmid at low temperature (16°C) with 150 rpm (Supplementary Figure S2).

Under the optimum condition, the significant enhancement of the expression level of the GDF8 fusion proteins was identified for all three novel vectors (Figures 8B–D). However, without the Vasp derivate sequences, GDF8 cannot be detected by SDS-PAGE, which was consistent with the previous observation (Gao et al., 2020). Notably, the coexistence of *Bt*TF and the Vasp-related sequences (VN, VNH, or V3N) made the GDF8 soluble, albeit in a small percentage. The triple-arranged VN (V3N) was unable to improve the expression level of GDF8 further compared to the single one. Compared to the common solubility enhancer tags, such as MBP, Trx, and GST, the Vasp and its variant VN brought out a better production yield for eGFP (Supplementary Figure S3).

A total of three proteins of *Setaria italica* including the cytochrome P450 71A1-like (71A1), ent-cassadiene C2-hydroxylase (ECH), and cinnamate beta-D-glucosyltransferase (CGT) were also expressed successfully in soluble form by the VNhte-*Bt*TF cassette (Supplementary Figure S4). Additionally, when located at the C-terminal of the recombinant proteins, VN failed to enhance the expression level of GDF8 and ECH proteins (Supplementary Figure S5).

### *Ec*TF Cannot Improve the Solubility of the VN Guided Recombinant Proteins

Trigger factor of *E. coli* (*Ec*TF) has a low identity with *Bt*TF (29.4%, Supplementary Figure S6). Therefore, the effect of *Ec*TF encoded by the extra chaperone plasmid pTf16 on the solubility of the fusion proteins VNhteGDF8, V3NhteGDF8, and VeGFP was investigated. Notably, in the VNhte-*Bt*TF-like cassettes, the rapid expression of the recombinant proteins and *Bt*TF was almost synchronous (Supplementary Figure S1) after adding IPTG. The pTf16 involved dual-plasmid system made it easy to produce the recombinant proteins and *Ec*TF asynchronously. The results showed that, when the host cells only harbored the pTf16 plasmid, the production of *Ec*TF was high after induction of L-arabinose. The coexistence with the second plasmid significantly reduced the accumulation of *Ec*TF, but the products can also be detected by SDS-PAGE analysis. Unfortunately, the extra produced *Ec*TF molecules were unable to enhance the solubility of VeGFP, VNhteGDF8, and V3NhteGDF8 (Supplementary Figure S7), which revealed the different specificities between *Ec*TF and *Bt*TF.

## Discussion

Due to the lack of the recognition site of signal peptidase, the length of Vsp has not been determined precisely. This study used the longest version (33 AAs) of Vasp to develop a novel protein expression system. The result showed that Vasp exerted a positive effect on the expression level of eGFP. Previously, we reported a novel fusion tag Iasp and proposed new insights into the abundant SPs of natural secretory proteins that would be an ideal resource for fusion tags (Gao et al., 2020). This study provided another example. However, the hydrophobic region of SPs negatively affected the thermodynamic stability of the cargo proteins, leading to the formation of inclusion bodies (Beena et al., 2004; Krishnan et al., 2009; Kulothungan et al., 2009; Singh et al., 2013). This drawback cannot be ignored unless the kind of the tags used was similar to the IB-tag, such as the SP of torA (Jong et al., 2017). This study identified two ways to at least partially avoid or solve the problem.

The N-region of Vasp (VN) could be considered as the polycationic tag harboring two lysine residues and one arginine residue. The net positive charged VN enhanced the expression level of eGFP with a slight compromise in its solubility. The polycationic tags containing the polylysine or polyarginine residues have been used as the protein solubility enhancers for almost three decades (reviewed in Paraskevopoulou and Falcone, 2018). The net charges are positively associated with the solubilizing effect and the polyarginine tags perform better than the polylysine tags, especially those located at the C-terminus of the partner protein. Unfortunately, the C-terminal location of VN failed to enhance the expression level of GDF8 and ECH proteins. Interestingly, the improvement in expression levels on recombinant proteins of most polycationic tags was negligible, such as diarginine (R2), hexaarginine (R6), decaarginine (R10), and decalysine (K10) (Jung et al., 2011). Since almost all of the classic SPs are comprised of a net positively charged region at the N-terminus (Owji et al., 2018), there must be a large number of VN-like candidates that would be used as the production yield and solubility dual-enhancers directly.

The negative impact of the regions of Vasp on the solubility of eGFP cannot be neutralized by *Ec*SecB, a common chaperone in the Sec pathway (Chatzi et al., 2013). *Ec*SecB also assists in the folding of some cytosolic clients such as barnase and MBP (Randall and Hardy, 2002; Ullers et al., 2004). *Ec*SecA plays a crucial role in transferring the preprotein into the inner membrane channel formed by the *Ec*SecYEG complex (Eser and Ehrmann, 2003). This protein binds to either the SPs or the mature domains of the client proteins and even interacts with the nascent polypeptides without the participation of the *Ec*SecB or *Ec*TF (Huber et al., 2017). The roles and their action orders of the crucial chaperones involved in the Sec pathway are intricate but employ different routes to the same destination (summarized in De Geyter et al., 2020). Briefly, the nascent peptides are recognized and bound by *Ec*SecB or *Ec*SecA. The binding prevents the aggregation of the client proteins and facilitates the next interactions. For instance, the *Ec*SecB:client complex targets the free or bound *Ec*SecA on *Ec*SecYEG translocon directly. The binding of *Ec*SecA to the nascent peptide was also reported, and the *Ec*SecA:client will be anchored by the *Ec*SecYEG translocon directly or after combining with *Ec*SecB. CsaA is the counterpart of SecB in many gram-positive bacteria, such as *Bacillus subtilis* and *Bt*, as well as in most of archaea (Müller et al., 2000a,b; Sala et al., 2013). In *B. subtilis, Bs*CsaA is a replacement of SecB to transport some preproteins such as ProOmpA and PrePhoB. In the present study, the effect of *Bt*CsaA on the VeGFP was investigated, but no visible change in the client solubility was obtained. Neither *Ec*SecB nor *Bt*SecA was able to prevent the products of VeGFP from aggregation. To exclude the possible interference resulting from the interaction between *Ec*SecB and *Ec*SecA *in vivo*, the performances of the variants of *Ec*SecB including SecB7577 and SecB142, as well as their combination SecB142-7577, were tested. Unfortunately, all of them also failed to keep the VeGFP soluble.

There should be another interaction model accounting for the translocation of Vip3. TF is a versatile chaperone, which either participates in the folding processes or anti-aggregation of several proteins (Patzelt et al., 2001; Bhandari and Houry, 2015; Saio et al., 2018; De Geyter et al., 2020), or surprisingly regulates the degradation of some newly synthesized proteins (Rizzolo et al., 2021). More importantly, it is a crucial chaperone in the Sec pathway. Recently, the following TF-associated models were elaborated in *E*. *coli* (Müller et al., 2000a; De Geyter et al., 2020): (1) TF-only model, named by the present study: The interaction of the client protein with TF (TF:client) is enough for settling down on the SecYEG-SecA translocase; (2) TF:bound-SecB model: The TF:client targets the translocase bound with the SecB already; (3) TF:free-SecB model: the TF:client is recognized and bound by the free SecB in the cytosol, and afterward, the triple complex is transported to the translocase; and (4) free-SecB:TF model: The SecB:client combination described above can interact with TF prior to reaching the location at the translocase. The contribution of *Bt*TF to the solubilization of VeGFP and its variants supported the models and implicated that the combination between Vasp and *Bt*TF would be preferential. It is important to note that TF is also associated with the ribosome located near the exit tunnel and plays a crucial role in the *de novo* folding of many cytosolic proteins by interaction with the nascent peptides (reviewed in Bhandari and Houry, 2015). Interestingly, *Ec*TF, which shares a low identity with *Bt*TF, did not affect the solubility of Vasp or its variants guided recombinant proteins. The interactions between Vasp or its variants and *Bt*TF are worthy of further investigation.

In conclusion, each region of Vasp could be used as a fusion tag to enhance the production yield of eGFP. This may promote a new approach to be developed for the natural secretory peptides. The corresponding region(s) of the Vasp in the fusion proteins also attracted the interaction with *Bt*TF, leading to the higher expression without compromise of the solubility. The solubility enhancement effects of a given fusion tag vary significantly depending on the cargo proteins *per se*, and unfortunately, the variations are not predictable so far. Therefore, the coexpression of the region(s) of Vasp, especially the VN harboring less hydrophobic residues, and *Bt*TF would be an ideal and predictable method to enhance the production yield and solubility of recombinant proteins simultaneously in the prokaryotic expression system.

## Data Availability Statement

The original contributions presented in the study are included in the article/Supplementary Material, further inquiries can be directed to the corresponding author/s.

## Author Contributions

CX, XW, and JG conceived of the presented idea and planned the experiments. JG, CO, JZ, and CX analyzed the data. JG, CO, XW, and CX prepared the manuscript. CO, JZ, YH, QG, TZ, and XL constructed the vectors and analyzed the protein expression. CO, JZ, and XL investigated fluorescent intensity. JG, CO, JZ, and MD took photographs with a confocal microscope. All authors reviewed the manuscript.

## Funding

This work was supported by Shanxi Key Laboratory of Minor Crops Germplasm Innovation and Molecular Breeding, Shanxi Agricultural University (Project 202105D121010) and the National Natural Science Foundation of China (Project 31601690).

## Conflict of Interest

The authors declare that the research was conducted in the absence of any commercial or financial relationships that could be construed as a potential conflict of interest.

## Publisher's Note

All claims expressed in this article are solely those of the authors and do not necessarily represent those of their affiliated organizations, or those of the publisher, the editors and the reviewers. Any product that may be evaluated in this article, or claim that may be made by its manufacturer, is not guaranteed or endorsed by the publisher.
