# Supplementary material for: Coexpressing the Signal Peptide of Vip3A and the Trigger Factor of Bacillus thuringiensis Enhances the Production Yield and Solubility of eGFP in Escherichia coli
Source: Front Microbiol. 2022 Jul 18;13:892428. doi: 10.3389/fmicb.2022.892428 (PMC9342664; doi:10.3389/fmicb.2022.892428)
Supplement: Supplementary file 1 [file Data_Sheet_1.docx]

Supplementary Material

**Supplementary Table S1 Plasmids and strains used in this work**

| **Plasmid** | **Characteristics** | **Source** |
| --- | --- | --- |
| pHT304 | Amp^r^, Erm^r^, *Bacillus*-*E.coli* shuttle vector | (Arantes and Lereclus, 1991) |
| p304ΔSacⅠ | pHT304 without *Sac*Ⅰ recognition site | (Gao et al., 2020) |
| pAc-eGFP | p304ΔSacⅠ, *P_ac_*-*egfp*-*T_ac_* expression cassette | (Gao et al., 2020) |
| pAc-IeGFP | p304ΔSacⅠ, *P_ac_-Iegfp-T_ac_* expression cassette | (Gao et al., 2020) |
| pAc-VeGFP | p304ΔSacⅠ, *P_ac_*-*Vegfp*-*T_ac_* expression cassette | This work |
| pET28aDel | Kan^r^, pET28a without the sequence between RBS and *Bam*H Ⅰ | (Gao et al., 2011) |
| p28aD-eGFP | pET28aDel, *P_T7_-egfp-T_T7_* expression cassette | (Gao et al., 2020) |
| p28aD-VeGFP | pET28aDel, *P_T7_-Vegfp-T_T7_* expression cassette | This work |
| p28aD-VNeGFP | pET28aDel, *P_T7_-VNegfp-T_T7_* expression cassette | This work |
| p28aD-VNHeGFP | pET28aDel, *P_T7_-VNHegfp-T_T7_* expression cassette | This work |
| p28aD-VNCeGFP | pET28aDel, *P_T7_-VNCegfp-T_T7_* expression cassette | This work |
| p28aD-VHeGFP | pET28aDel, *P_T7_-VHegfp-T_T7_* expression cassette | This work |
| p28aD-VHCeGFP | pET28aDel, *P_T7_-VHCegfp-T_T7_* expression cassette | This work |
| p28aD-VCeGFP | pET28aDel, *P_T7_-VCegfp-T_T7_* expression cassette | This work |
| p28aD-VeGFP-EcSecB | pET28aDel, *P_T7_-Vegfp-P_BtCsaA_-EcSecB-T_T7_* expression cassette | This work |
| p28aD-VeGFP-SecB7577 | pET28aDel, *P_T7_-Vegfp-P_BtCsaA_-SecB7577-T_T7_* expression cassette | This work |
| p28aD-VeGFP-SecB142 | pET28aDel, *P_T7_-Vegfp-P_BtCsaA_-SecB142-T_T7_* expression cassette | This work |
| p28aD-VeGFP-SecB142-7577 | pET28aDel, *P_T7_-Vegfp-P_BtCsaA_-SecB142-7577-T_T7_* expression cassette | This work |
| p28aD-VeGFP-BtCsaA | pET28aDel, *P_T7_-Vegfp-P_BtCsaA_-BtCsaA-T_T7_* expression cassette | This work |
| p28aD-VeGFP-BtSecA | pET28aDel, *P_T7_-Vegfp-P_BtCsaA_-BtSecA-T_T7_* expression cassette | This work |
| p28aD-VeGFP-BtTF | pET28aDel, *P_T7_-Vegfp-P_BtCsaA_-Bttig-T_T7_* expression cassette | This work |
| p28aD-BtTF | pET28aDel, *P_T7_-Bttig-T_T7_* expression cassette | This work |
| p28aD-VNeGFP-BtTF | pET28aDel, *P_T7_-VNegfp-P_BtCsaA_-Bttig-T_T7_* expression cassette | This work |
| p28aD-VNHeGFP-BtTF | pET28aDel, *P_T7_-VNHegfp-P_BtCsaA_-Bttig-T_T7_* expression cassette | This work |
| p28aD-VNCeGFP-BtTF | pET28aDel, *P_T7_-VNCegfp-P_BtCsaA_-Bttig-T_T7_* expression cassette | This work |
| p28aD-VHeGFP-BtTF | pET28aDel, *P_T7_-VHegfp-P_BtCsaA_-Bttig-T_T7_* expression cassette | This work |
| p28aD-VHCeGFP-BtTF | pET28aDel, *P_T7_-VHCegfp-P_BtCsaA_-Bttig-T_T7_* expression cassette | This work |
| p28aD-VCeGFP-BtTF | pET28aDel, *P_T7_-VCegfp-P_BtCsaA_-Bttig-T_T7_* expression cassette | This work |
| p28aD2-T-eGFP | pET28aDel2, *P_T7_*-*Trx*-*egfp*-*T_T7_* expression cassette | (Gao et al., 2020) |
| p28aD2-G-eGFP | pET28aDel2, *P_T7_*-*GST*-*egfp*-*T_T7_* expression cassette | This work |
| p28aD2-M-eGFP | pET28aDel2, *P_T7_*-*MBP*-*egfp*-*T_T7_* expression cassette | (Gao et al., 2020) |
| p28aD2-GDF8 | pET28aDel2, *P_T7_*-*gdf8*-*T_T7_* expression cassette | (Gao et al., 2020) |
| p28aD-VNhteGDF8-BtTF | pET28aDel, *P_T7_-VNhtegdf8-P_BtCsaA_-Bttig-T_T7_* expression cassette | This work |
| p28aD-VNhteGDF8 | pET28aDel, *P_T7_-VNhtegdf8-T_T7_* expression cassette | This work |
| p28aD-VNHhteGDF8-BtTF | pET28aDel, *P_T7_-VNHhtegdf8-P_BtCsaA_-Bttig-T_T7_* expression cassette | This work |
| p28aD-VNHhteGDF8 | pET28aDel, *P_T7_-VNHhtegdf8-T_T7_* expression cassette | This work |
| p28aD-V3NhteGDF8-BtTF | pET28aDel, *P_T7_-V3Nhtegdf8-P_BtCsaA_-Bttig-T_T7_* expression cassette | This work |
| p28aD-V3NhteGDF8 | pET28aDel, *P_T7_-V3Nhtegdf8-T_T7_* expression cassette | This work |
| p28aD-GDF8VN | pET28aDel, *P_T7_*-*gdf8VN-T_T7_* expression cassette | This work |
| p28aD-GDF8VN-BtTF | pET28aDel, *P_T7_*-*gdf8VN-P_BtCsaA_-Bttig-T_T7_* expression cassette | This work |
| p28aD-VNhte71A1-BtTF | pET28aDel, *P_T7_-VNhte71A1-P_BtCsaA_-Bttig-T_T7_* expression cassette | This work |
| p28aD-71A1-BtTF | pET28aDel, *P_T7_-71A1-P_BtCsaA_-Bttig-T_T7_* expression cassette | This work |
| p28aD-VNhteECH-BtTF | pET28aDel, *P_T7_-VNhteECH-P_BtCsaA_-Bttig-T_T7_* expression cassette | This work |
| p28aD-ECH-BtTF | pET28aDel, *P_T7_-ECH-P_BtCsaA_-Bttig-T_T7_* expression cassette | This work |
| p28aD-ECHVN | pET28aDel, *P_T7_*-*ECHVN-T_T7_* expression cassette | This work |
| p28aD-ECHVN-BtTF | pET28aDel, *P_T7_*-*ECHVN-P_BtCsaA_-Bttig-T_T7_* expression cassette | This work |
| p28aD-VNhteCGT-BtTF | pET28aDel, *P_T7_-VNhteCGT-P_BtCsaA_-Bttig-T_T7_* expression cassette | This work |
| p28aD-CGT-BtTF | pET28aDel, *P_T7_-CGT-P_BtCsaA_-Bttig-T_T7_* expression cassette | This work |
| pTf16 | Cm^r^, *P_araB_*-*Ectig* expression cassette, pACYC *ori* | Takara |
| **Strain** | **Genotype** | **Source** |
| *E. coli* |  |  |
| TG1 | K12Δ(lac-pro), *supE*, *thi*, *hsdD5*, *F^–^* [*traD36 proAB^+^ lacI^q^* *lacZΔM15*] | (Gibson, 1984) |
| MC4100 | F^–^, *ΔlacU169*, *araD139*, *rpsL150*, *relA1*, *ptsF*, *rbsR*, *flbB5301* | (Casadaban and Cohen, 1979) |
| BL21-star (DE3) | F^–^ *omp*T *hsd*S_B_ (r_B_^-^, m_B_^-^) *galdcmrne*131 (DE3) | Thermo Fisher Scientific |
| MC304 | MC4100 harboring pHT304 plasmid | This work |
| MCAc-eGFP | MC4100 transformed with pAc-eGFP | This work |
| MCAc-IeGFP | MC4100 harboring pAc-IeGFP plasmid | This work |
| MCAc-VeGFP | MC4100 harboring pAc-VeGFP plasmid | This work |
| BL28aD | BL21-star (DE3) transformed with pET28aDel | This work |
| BL28-eGFP | BL21-star (DE3) transformed with p28aD-eGFP | This work |
| BL28-VeGFP | BL21-star (DE3) transformed with p28aD-VeGFP | This work |
| BL28-VNeGFP | BL21-star (DE3) transformed with p28aD-VNeGFP | This work |
| BL28-VNHeGFP | BL21-star (DE3) transformed with p28aD-VNHeGFP | This work |
| BL28-VNCeGFP | BL21-star (DE3) transformed with p28aD-VNCeGFP | This work |
| BL28-VHeGFP | BL21-star (DE3) transformed with p28aD-VHeGFP | This work |
| BL28-VHCeGFP | BL21-star (DE3) transformed with p28aD-VHCeGFP | This work |
| BL28-VCeGFP | BL21-star (DE3) transformed with p28aD-VCeGFP | This work |
| BL28-VeGFP-EcSecB | BL21-star (DE3) transformed with p28aD-VeGFP-EcSecB | This work |
| BL28-VeGFP-SecB7577 | BL21-star (DE3) transformed with p28aD-VeGFP-SecB7577 | This work |
| BL28-VeGFP-SecB142 | BL21-star (DE3) transformed with p28aD-VeGFP-SecB142 | This work |
| BL28-VeGFP-Sec142-7577 | BL21-star (DE3) transformed with p28aD-VeGFP-Sec142-7577 | This work |
| BL28-VeGFP-BtSecA | BL21-star (DE3) transformed with p28aD-VeGFP-BtSecA | This work |
| BL28-VeGFP-BtCsaA | BL21-star (DE3) transformed with p28aD-VeGFP-BtCsaA | This work |
| BL28-VeGFP-BtTF | BL21-star (DE3) transformed with p28aD-VeGFP-BtTF | This work |
| BL21-BtTF | BL21-star (DE3) transformed with p28aD-BtTF | This work |
| BL28-VNeGFP-BtTF | BL21-star (DE3) transformed with p28aD-VNeGFP-BtTF | This work |
| BL28-VNHeGFP-BtTF | BL21-star (DE3) transformed with p28aD-VNHeGFP-BtTF | This work |
| BL28-VNCeGFP-BtTF | BL21-star (DE3) transformed with p28aD-VNCeGFP-BtTF | This work |
| BL28-VHeGFP-BtTF | BL21-star (DE3) transformed with p28aD-VHeGFP-BtTF | This work |
| BL28-VHCeGFP-BtTF | BL21-star (DE3) transformed with p28aD-VHCeGFP-BtTF | This work |
| BL28-VCeGFP-BtTF | BL21-star (DE3) transformed with p28aD-VCeGFP-BtTF | This work |
| BL28-T-eGFP | BL21-star (DE3) transformed with p28aD2-T-eGFP | This work |
| BL28-G-eGFP | BL21-star (DE3) transformed with p28aD2-G-eGFP | This work |
| BL28-M-eGFP | BL21-star (DE3) transformed with p28aD2-M-eGFP | This work |
| BL28-GDF8 | BL21-star (DE3) transformed with p28aD2-GDF8 | This work |
| BL28-VNhteGDF8-BtTF | BL21-star (DE3) transformed with p28aD-VNhteGDF8-BtTF | This work |
| BL28-VNhteGDF8 | BL21-star (DE3) transformed with p28aD-VNhteGDF8 | This work |
| BL28-VNHhteGDF8-BtTF | BL21-star (DE3) transformed with p28aD-VNHhteGDF8-BtTF | This work |
| BL28-VNHhteGDF8 | BL21-star (DE3) transformed with p28aD-VNHhteGDF8 | This work |
| BL28-V3NhteGDF8-BtTF | BL21-star (DE3) transformed with p28aD-V3NhteGDF8-BtTF | This work |
| BL28-V3NhteGDF8 | BL21-star (DE3) transformed with p28aD-V3NhteGDF8 | This work |
| BL28-GDF8VN | BL21-star (DE3) transformed with p28aD-GDF8VN | This work |
| BL28-GDF8VN-BtTF | BL21-star (DE3) transformed with p28aD-GDF8VN-BtTF | This work |
| BL28-VNhte71A1-BtTF | BL21-star (DE3) transformed with p28aD-VNhte71A1-BtTF | This work |
| BL28-71A1-BtTF | BL21-star (DE3) transformed with p28aD-71A1-BtTF | This work |
| BL28-VNhteECH-BtTF | BL21-star (DE3) transformed with p28aD-VNhteECH-BtTF | This work |
| BL28-ECH-BtTF | BL21-star (DE3) transformed with p28aD-ECH-BtTF | This work |
| BL28-ECHVN | BL21-star (DE3) transformed with p28aD-ECHVN | This work |
| BL28-ECHVN-BtTF | BL21-star (DE3) transformed with p28aD-ECHVN-BtTF | This work |
| BL28-VNhteCGT-BtTF | BL21-star (DE3) transformed with p28aD-VNhteCGT-BtTF | This work |
| BL28-CGT-BtTF | BL21-star (DE3) transformed with p28aD-CGT-BtTF | This work |
| BL-pTf16 | BL21-star (DE3) transformed with pTf16 | This work |
| BL28D-pTf16 | BL21-star (DE3) transformed with pET28aDel and pTf16 | This work |
| BL28D-VeGFP-pTf16 | BL21-star (DE3) transformed with p28aD-VeGFP and pTf16 | This work |
| BL28D-VNhteGDF8-pTf16 | BL21-star (DE3) transformed with p28aD-VNhteGDF8 and pTf16 | This work |
| BL28D-V3NhteGDF8-pTf16 | BL21-star (DE3) transformed with p28aD-V3NhteGDF8 and pTf16 | This work |

**Supplementary Table S2 Primers used in this work**

| **Primer** | **Sequence (5**′**-3**′**)** |
| --- | --- |
| VEGFP-F | CAGTGGATCCACCATGAACAAGAATAATACTAA |
| VEGFP-fuF | CTGGTATCAAAGACATTATGATGGGTAAAGGAGAAGAACT |
| VEGFP-fuR | AGTTCTTCTCCTTTACCCATCATAATGTCTTTGATACCAG |
| I/EGFP-R | GACTCTCGAGTTTGTATAGTTCATCCATGCCA |
| 304I/EGFP-R | GAGCTCTTAGTGGTGGTGGTGGTGGTGCT |

Restriction sites were underline.

**Supplementary Table S3 The predicted secretory peptide sequences of Vip3A proteins and the corresponding members**

| **Represent Gene** | **Vsp sequence** | **Genes** |
| --- | --- | --- |
| Vip3Aa1 | MNKNNTKLSTRALPSFIDYFNGIYGFATGIKDI | Vip3Aa10, Vip3Aa11, Vip3Aa12, Vip3Aa13, Vip3Aa14, Vip3Aa15, Vip3Aa16, Vip3Aa18, Vip3Aa19, Vip3Aa2, Vip3Aa20, Vip3Aa21, Vip3Aa22, Vip3Aa24, Vip3Aa25, Vip3Aa26, Vip3Aa27, Vip3Aa28, Vip3Aa33, Vip3Aa34, Vip3Aa35, Vip3Aa36, Vip3Aa37, Vip3Aa4, Vip3Aa42, Vip3Aa43, Vip3Aa44, Vip3Aa46, Vip3Aa47, Vip3Aa49, Vip3Aa5, Vip3Aa52, Vip3Aa55, Vip3Aa57, Vip3Aa58, Vip3Aa59, Vip3Aa6, Vip3Aa61, Vip3Aa62, Vip3Aa64, Vip3Aa65, Vip3Aa7, Vip3Aa8, Vip3Aa9, Vip3Ag12, Vip3Ag14, Vip3Ag7, Vip3Ag8, Vip3Ah1 |
| Vip3Aa45 | MNKNNTKLSTRALPSFIDYFNGIYGFATGIKDI |  |
| Vip3Aa48 | MNKNNTKLSTRALPSFIDYFNGVYGFATGIKDI |  |
| Vip3Aa53 | MNKNNTKLNTRALPSFIDYFNGIYGFATGIKDI |  |
| Vip3Aa17 | MNKNNTKLNARALPSFIDYFNGIYGFATGIKDI | Vip3Ag1, Vip3Ag10, Vip3Ag15, Vip3Ag4, Vip3Ag6, Vip3Ca1, Vip3Ca3, Vip3Ca4 |
| Vip3Aa40 | MNKNNTKLSARALPSFIDYFNGIYGFATGIKDI | Vip3Aa56, Vip3Af4, Vip3Ag2, Vip3Ag5, Vip3Bb3 |
| Vip3Ai1 | MNMNKNNTKLSARALPSFIDYFNGIYGFATGIKDI |  |
| Vip3Aa54 | MNKNNTKLSTRALPSFIDYFNGIYGFTTGIKDI |  |
| Vip3Aa23 | MNKNNTKLSTRALPGFIDYFNGIYGFATGIKDI |  |
| Vip3Aa50 | MTKNNTKLSTRALPSFIDYFNGIYGFATGIKDI |  |
| Vip3Aa38 | MNMNNTKLNARALPSFIDYFNGIYGFATGIKDI | Vip3Aa39, Vip3Aa51, Vip3Ab1, Vip3Ab2, Vip3Ad1, Vip3Ae1, Vip3Af1, Vip3Af2, Vip3Af3, Vip3Ag3, Vip3Ba1, Vip3Ba2, Vip3Ca2 |
| Vip3Bb1 | MNNTKLNARALPSFIDYFNGIYGFATGIKDI | Vip3Bb2 |
| Vip3Aa63 | MNMNNTKLSARALPSFIDYFNGIYGFATGIKDI |  |
| Vip3Aa41 | MNMNNTKLSARALPSLIDYFNGIYGFATGIKDI |  |
| Vip3Aa60 | MNMNNTKLSTRALPSFIDYFNGIYGFATGIKDI |  |
| Vip3Ah2 | MNMNNTKLSTRALPSFIDYFNGIYGFATGIKDI |  |
| Vip3Ad2 | MNMNNAKLNARALPSFIDYFNGIYGFATGIKDI |  |
| Vip3Ad4 | MNMNNTKLNARALPSFIDYFNGIYGFAIGIKDI | Vip3Ad6 |
| Vip3Ad5 | MNMNNAKLNARALPSFIDYFNGIYGFAIGIKDI |  |
| Vip3Ad3 | MNKNNTKLNARALPSFIDYFNGIYGFAIGIKDI | Vip3Ag11, Vip3Ag13, Vip3Ag9, Vip3Aj1, Vip3Aj2 |
| Vip3Bc1 | MVQKWMQRMIIVDNNKLNVRALPSFIDYFNGIYGFATGIKDI |  |
| Vip3Ac1 | MQKNNKLSVKALPSFIDYFNGIYGFATGIKDI |  |
| Vip3Aa30 | MNKNNTKLSHVVVISFIDYFNGIYGFATGIKDI |  |
| Vip3Aa31 | MNKNNTKLSNVNELSSLSDYFNGIYGFATGIKDI |  |
| Vip3Aa32 | MNKNNTKLSKNELSRLSDYFNGIYGFATGIKDI |  |
| Vip3Aa29 | MNKNNTKLSNFRCLYLVEYFNGIYGFATGIKDI |  |

**Supplementary Table S4 The related quantification of the fusion fluorescent proteins in *E. coli***

| **Protein** | **Relative content of replicates (%)** | | | **Mean** | **SEM** |
| --- | --- | --- | --- | --- | --- |
|  | **1** | **2** | **3** |  |  |
| eGFP | 1.00 | 1.00 | 1.00 | 1.00 | 0.00 |
| VeGFP | 2.45 | 3.28 | 2.75 | 2.83 | 0.24 |
| VNeGFP | 3.09 | 1.87 | 3.22 | 2.73 | 0.43 |
| VHeGFP | 2.70 | 1.94 | 3.20 | 2.61 | 0.37 |
| VCeGFP | 3.01 | 2.45 | 2.02 | 2.49 | 0.29 |
| VNHeGFP | 2.45 | 3.12 | 2.90 | 2.82 | 0.20 |
| VNCeGFP | 3.90 | 2.57 | 2.74 | 3.07 | 0.42 |
| VHCeGFP | 2.34 | 3.79 | 3.46 | 3.20 | 0.44 |
