# Supplementary material for: Coexpressing the Signal Peptide of Vip3A and the Trigger Factor of Bacillus thuringiensis Enhances the Production Yield and Solubility of eGFP in Escherichia coli
Source: Front Microbiol. 2022 Jul 18;13:892428. doi: 10.3389/fmicb.2022.892428 (PMC9342664; doi:10.3389/fmicb.2022.892428)
Supplement: Supplementary file 2 [file Data_Sheet_2.pdf]

Supplementary Material

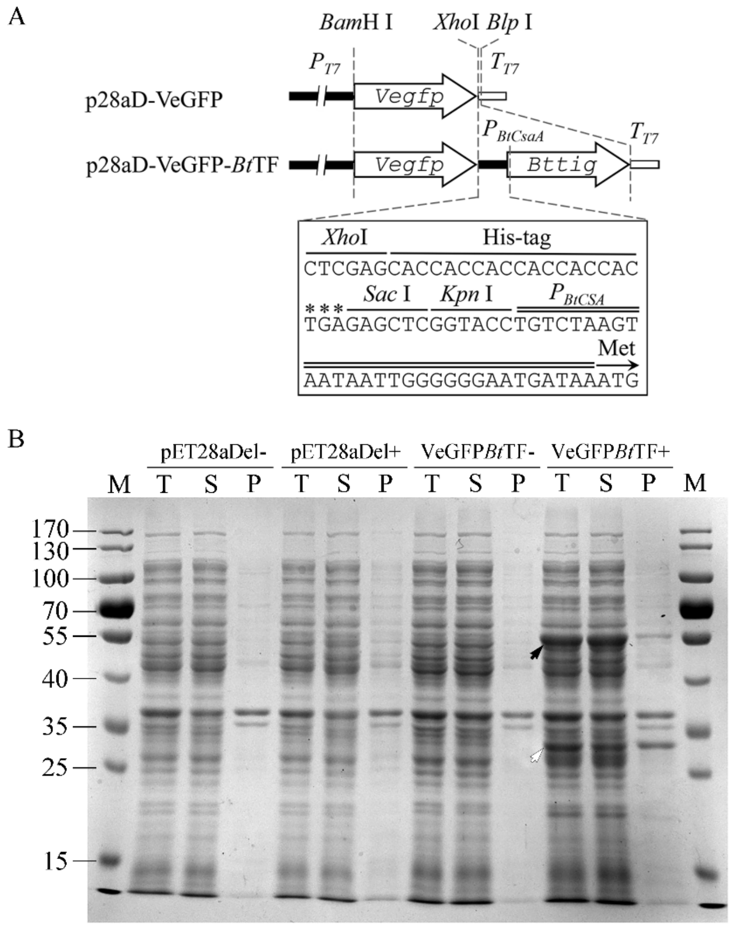

Supplementary Figure S1 Diagram of co-expression vector containing *Vegfp* and *Bttig* gene (A) and its expression results after induction (B). (A) The sequences between the *Vegfp* and *Bttig* genes were showed in the below box in which the asterisks (*) indicate the stop codon of *Vegfp* gene and the arrow indicates the initiation site of the chaperon gene. **(B)** The expression and solubility of the VeGFP and *Bt*TF in BL28-VeGFP-BtTF strain before (-) and after (+) induction. For each sample, the total proteins (T), the soluble component after cell lysis (S) and the precipitates (P) were loaded, respectively. Lane “M” is the molecular weight standards. The hollowed arrow indicates the VeGFP and the black arrow indicates the *Bt*TF.

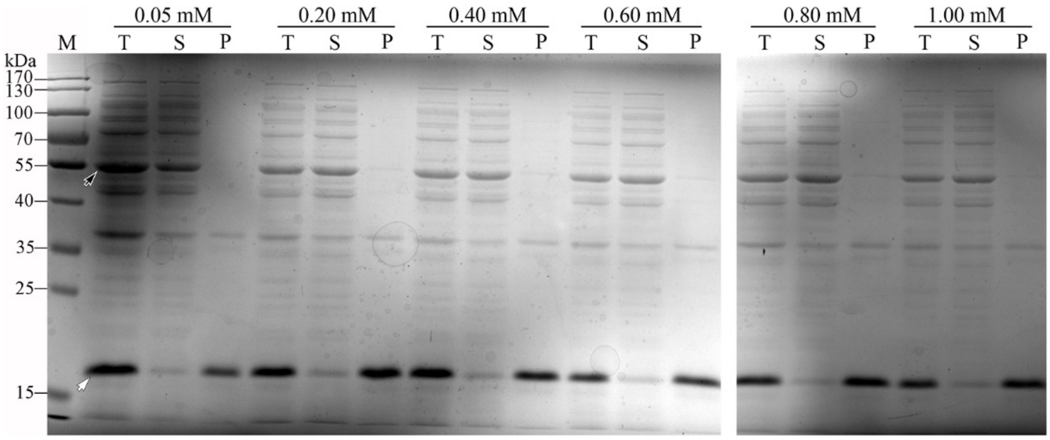

Supplementary Figure S2 The effect of the IPTG concentrations on the co-expression of VNhteGDF8 and *Bt*TF proteins. For each sample, the total proteins (T), the soluble component after cell lysis (S) and the precipitates (P) were loaded, respectively. Lane "M" is the molecular weight standards. The black arrow indicates the *Bt*TF and the hollowed arrow indicates the VNhteGDF8 (16.9 kDa).

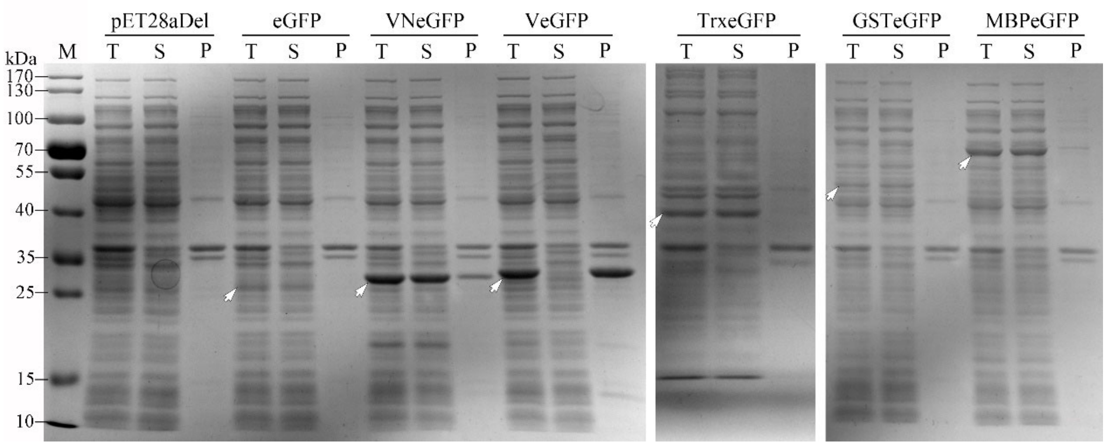

Supplementary Figure S3 Comparison of the Vasp and its derivate VN to three common solubility enhancer tags on the expression of eGFP. For each sample, the total proteins (T), the soluble component after cell lysis (S) and the precipitates (P) were loaded, respectively. Lane "M" is the molecular weight standards. The hollowed arrows indicate the recombinant proteins.

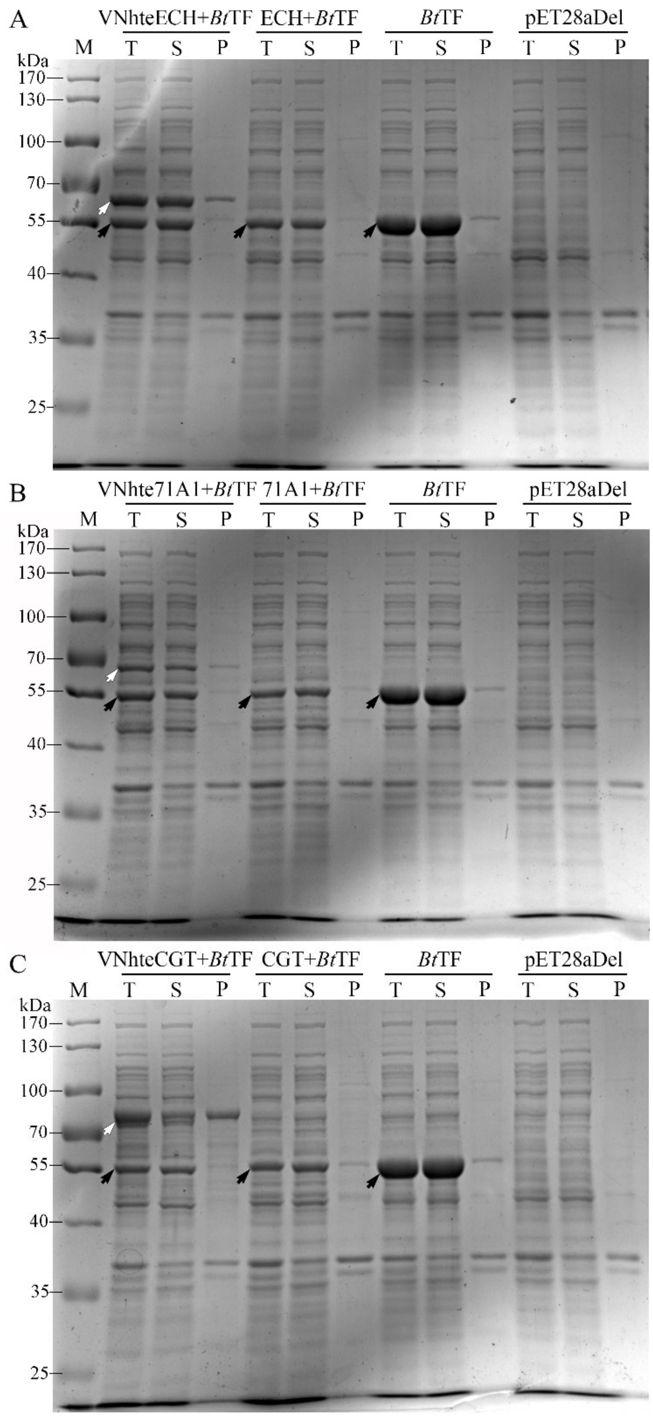

Supplementary Figure S4 The expression of ECH (A), 71A1 (B) and CGT (C) proteins using VNhte-*Bt*TF cassette. For each sample, the total proteins (T), the soluble component after cell lysis (S) and the precipitates (P) were loaded, respectively. Lane “M” is the molecular weight standards. The black arrows indicate the *Bt*TF and the hollowed arrows indicate the recombinant proteins.

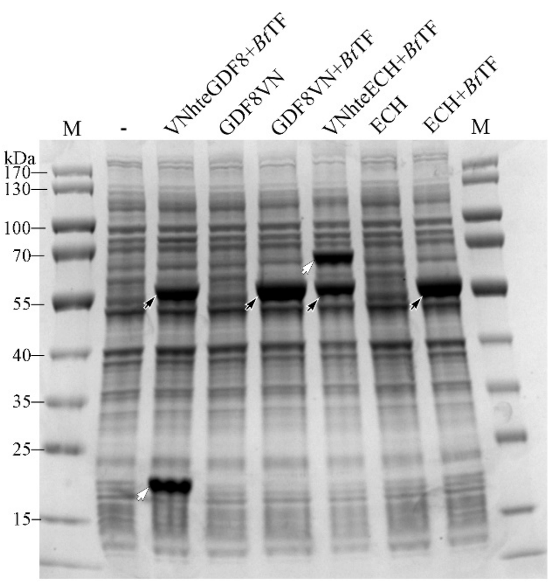

Supplementary Figure S5 The expression of recombinant proteins when VN located at the C-terminal of GDF8 and ECH. The SDS-PAGE were conducted in 4-20% gradient gel with MOPS buffer. For each sample, the total proteins of host cells were loaded. Lane “M” is the molecular weight standards. The black arrows indicate the *Bt*TF and the hollowed arrows indicate the recombinant proteins.

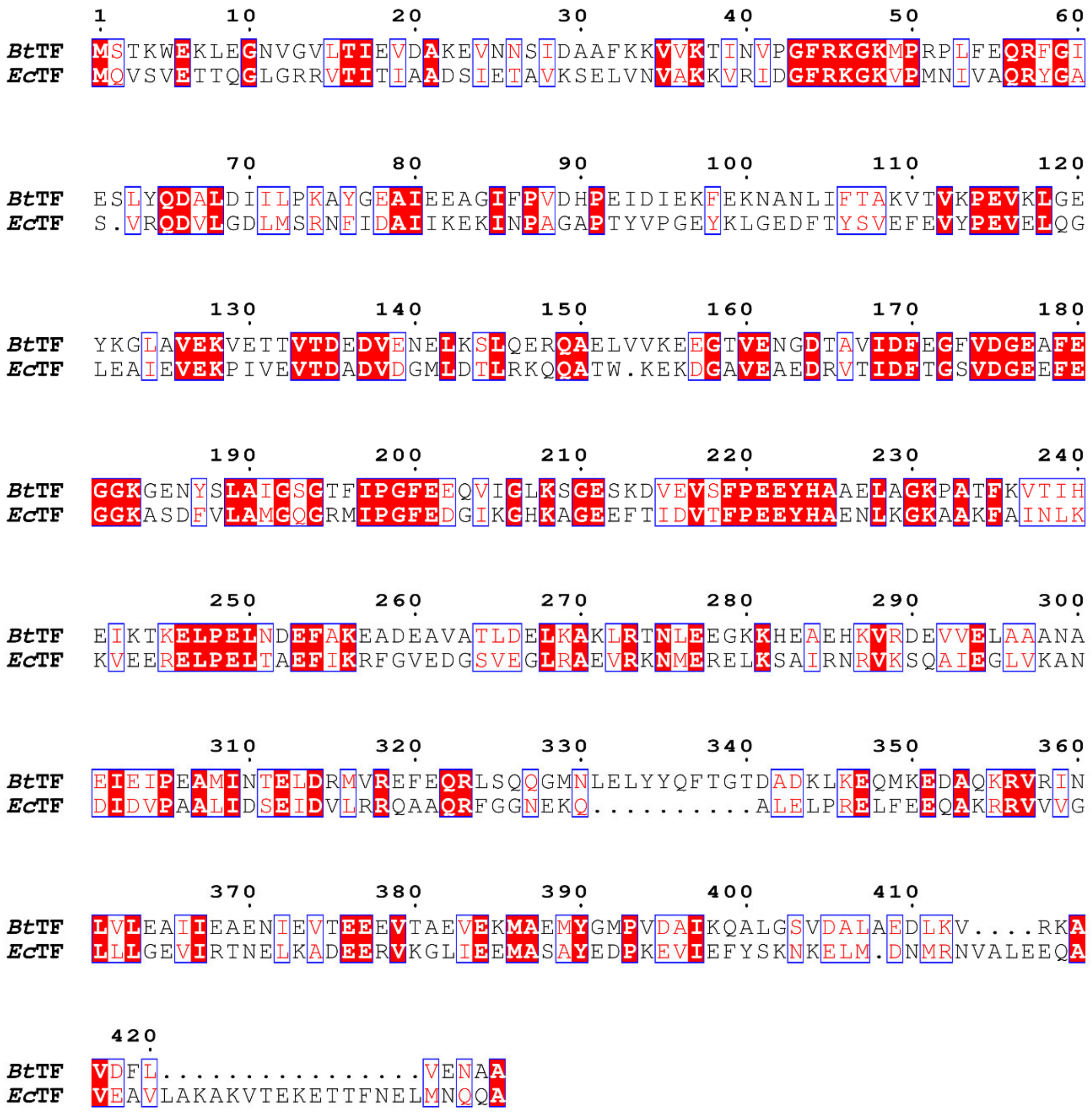

Supplementary Figure S6 The sequences alignment of *Bt*TF and *Ec*TF proteins.

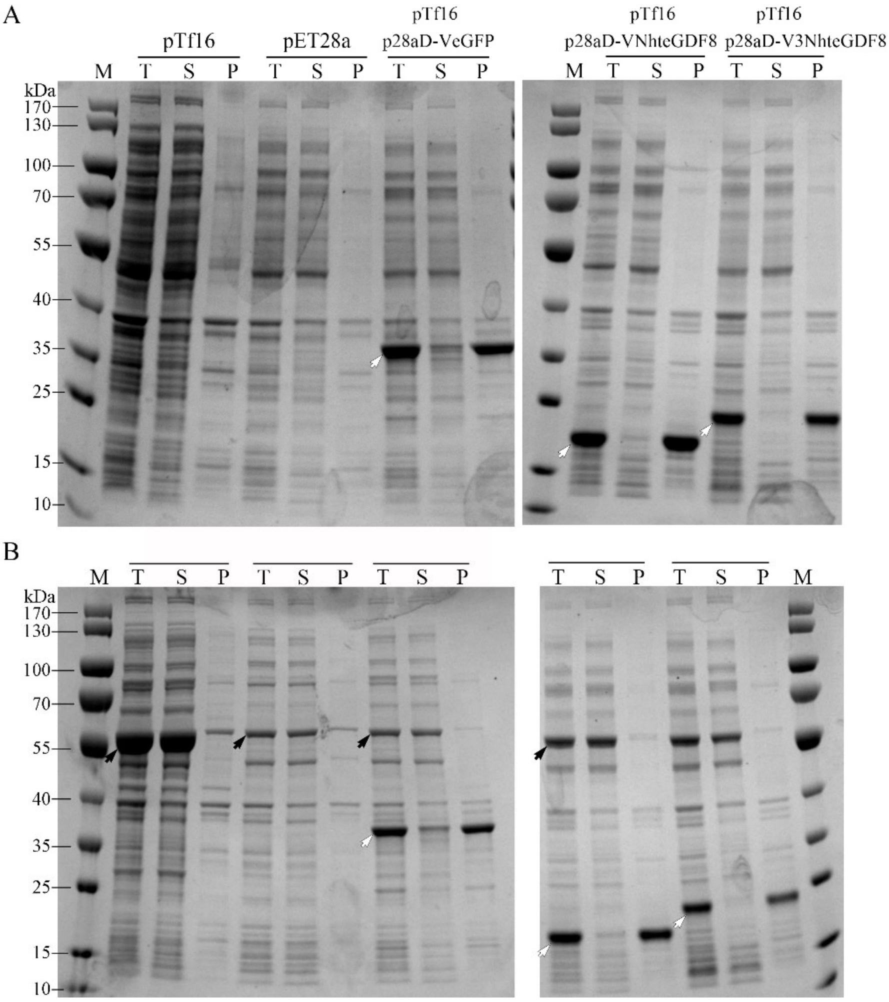

Supplementary Figure S7 The effect of *Ec*TF on the solubility of VeGFP, VNhteGDF8 and V3NhteGDF8 proteins. The SDS-PAGE were conducted in 4-20% gradient gel with MOPS buffer. For each sample, the total proteins (T), the soluble component after cell lysis (S) and the precipitates (P) were loaded, respectively. Lane “M” is the molecular weight standards. The black arrows indicate the *Ec*TF (48.2 kDa) and the hollowed arrows indicate the recombinant proteins.
